# A new electromechanical trainer for sensorimotor rehabilitation of paralysed fingers: A case series in chronic and acute stroke patients

**DOI:** 10.1186/1743-0003-5-21

**Published:** 2008-09-04

**Authors:** Stefan Hesse, H Kuhlmann, J Wilk, C Tomelleri, Stephen GB Kirker

**Affiliations:** 1 Klinik Berlin, Department Neurological Rehabilitation, Charité – University Medicine Berlin, Germany; 2 Addenbrooke's Rehabilitation Clinic, Cambridge University Hospitals NHS Foundation Trust, Cambridge, CB2 2QQ, UK

## Abstract

**Background:**

The functional outcome after stroke is improved by more intensive or sustained therapy. When the affected hand has no functional movement, therapy is mainly passive movements. A novel device for repeating controlled passive movements of paralysed fingers has been developed, which will allow therapists to concentrate on more complicated tasks. A powered cam shaft moves the four fingers in a physiological range of movement.

**Methods:**

After refining the training protocol in 2 chronic patients, 8 sub-acute stroke patients were randomised to receive additional therapy with the Finger Trainer for 20 min every work day for four weeks, or the same duration of bimanual group therapy, in addition to their usual rehabilitation.

**Results:**

In the chronic patients, there was a sustained reduction in finger and wrist spasticity, but there was no improvement in active movements. In the subacute patients, mean distal Fugl-Meyer score (0–30) increased in the control group from 1.25 to 2.75 (ns) and 0.75 to 6.75 in the treatment group (p < .05). Median Modified Ashworth score increased 0/5 to 2/5 in the control group, but not in the treatment group, 0 to 0. Only one patient, in the treatment group, regained function of the affected hand. No side effects occurred.

**Conclusion:**

Treatment with the Finger Trainer was well tolerated in sub-acute & chronic stroke patients, whose abnormal muscle tone improved. In sub-acute stroke patients, the Finger Trainer group showed small improvements in active movement and avoided the increase in tone seen in the control group. This series was too small to demonstrate any effect on functional outcome however.

## Introduction

The annual stroke incidence is approximately 180 patients per 100,000 inhabitants in the industrialized world. About 30% of the surviving patients suffer from a severe upper limb paresis with a non functional hand. The prognosis for regaining meaningful hand activity six months after stroke onset is poor [1]: this may partly be because current rehabilitation practice puts more emphasis on the compensatory use of the non-affected upper extremity [2].

Powered machines which can allow prolonged repetition of a controlled movement are a promising way of increasing the intensity of rehabilitation after stroke. Several devices, to treat wrist, elbow & shoulder movements, have been developed since the pioneering MIT-Manus in the early 1990s [3]. Randomized controlled trials show a convincing beneficial effect of robot-assisted upper limb treatment on the impairment of severely affected stroke patients [4-9].

There are fewer clinical reports of machine-assisted movement of paralysed fingers. The Rutgers Hand Masters I and II use pistons mounted inside the palm to move the fingers, with virtual reality to improve motivation. Chronic stroke patients improved range of motion, motor control and speed of the paretic fingers over several weeks of training, and the benefits were retained at follow-up [10,11].

With the Howard Hand Robot, pistons assist with patient initiated grasping and releasing movements around virtual or real objects. In moderately affected chronic stroke subjects, upper limb motor functions improved, and functional MRI revealed increased sensorimotor cortex activation during the grasping task which was not seen during a non-practiced task, supination/pronation [12].

Fischer et al assisted the finger extension of mildly affected stroke patients with the help of a powered orthosis. Following six weeks of training in reach-to-grasp of virtual and actual objects, patients' active motor performance had shown a moderate improvement [13].

The treatment of the plegic fingers after stroke is pertinent given their large cortical representation, the presumed competition between proximal and distal limb segments for plastic brain territory [14], and recent results from the MIT-group promoting earlier active treatment of distal limb [15]. Further, paresis-related immobilization seems to contribute to the development of long-term disabling finger flexor spasticity [16].

We have designed an electromechanical Finger Trainer to move individual fingers in a physiological range of movement. This article describes the device and reports its use in a small number of chronic and acute stroke patients with completely paralysed hands.

## Device

**Figure 1:**
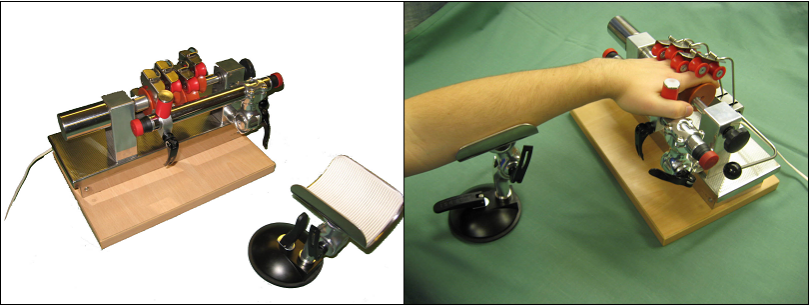
The Finger Trainer, "Reha-Digit", without a patient (left), and a left-hemiparetic patient practicing with the device (right).

The Finger Trainer, Reha-Digit, (figure 1) consists of four, mutually independent plastic rolls, each fixed eccentrically to the powered axle of the device, forming a cam-shaft. Each finger-roll can be repositioned & secured by turning a knob on the main axle, on the other end from the motor, to fit the size & range of movement of each individual finger.

The surface of each finger roll is concave, forming a gutter to maximise the contact area between finger & roll. Two smaller locking rollers, also concave, hold each finger against the larger finger roll. Each pair of locking rollers moves orthogonally to the axis of the finger roll, and an elastic spring pulls each pair of locking rollers towards the finger roller. These can be lifted out of the way when first positioning the hand & fingers in the device.

A spacing bar, parallel to the drive axle, holds the hand in the optimal position: a thumb stop may be used to provide additional stability. This can be moved to either side, to accommodate either the left or right hand. There are emergency-stop switches at each end of the spacing bar. The forearm can be stabilised at the correct angle & height on a gutter support.

A 24 V DC motor rotates the drive axle up to 30 times a minute through a clutch mechanism, which allows the axle to stop rotating if the hand goes into a powerful spasm. A vibration engine, situated under the base plate, provides small amplitude (2 mm) stimulation at a frequency which can be set between 0 to 30 Hz, by turning a knob. The device's weight is 7 kg, and its dimensions are 35 cm × 24 cm × 22 cm.

## Treatment

The patient sat comfortably on a chair with a backrest, with the device on a height-adjustable table in front of him. A therapist positioned the forearm on the arm support, placed the patients' four fingers II – V onto the cam shaft, and placed the thumb behind the spacing-bar or under the thumb-stop. The patient should not report any pain. In case of severe finger flexor spasticity, the therapist manually reduced the muscle tone before putting the hand in the device, and ultrasound contact gel could be applied to the fingers to diminish the friction between fingers and finger-rolls.

Initially, the rotation speed of the cam shaft and the vibration frequency were set at 20 rotations per minute and 20 Hz. After three minutes the treatment was interrupted in order to modify the treatment conditions regarding positioning, rotation speed and vibration frequency. The patient practised a total of 15 min with the device. The patients were instructed to concentrate on the movement of the paretic fingers and, if possible, to imagine that they themselves performed the finger movements.

To avoid saturation of the Meissner organs by continuous tactile dynamic stimulation of the finger tips by the revolving rolls, strips with different surface texture were attached to the inner surface of the concave roll of the index finger, and the patients were asked to discriminate between them. Patients with arthritis of the finger joints, soft tissue pain or hand swelling were excluded.

## Case series

### Chronic patients

Two male chronic stroke patients, aged 55 (#1) and 67 (#2) years, had suffered a supratentorial left hemisphere stroke resulting in a right hemiparesis 17 and 22 months before study onset. They participated in a comprehensive 4 week in-patient rehabilitation programme for chronic stroke patients. The rehab programme included 45 minutes each of Bobath orientated physiotherapy and occupational therapy every workday, of which upper limb rehabilitation made up about 15% of physiotherapy and 30% of occupational therapy time.

The patients did not take any oral muscle relaxants, and had no botulinum toxin injections in the preceding 3 months. Both were ambulatory and almost competent in the basic activities of daily living. The paretic upper extremity was severely affected, i.e. non-functional, and the fist was clenched due to severe wrist and finger flexor spasticity. The modified Ashworth scores (0–5) [17], was 4/5 for the fingers and 3/5 for wrist in both patients. The Fugl-Meyer Motor Score for the upper limb (FM, 0–66) [18], was 7 and 9 respectively. Pain, touch and position sensation, and two-point discrimination were unimpaired.

Initially, a therapist had to open the clenched fist before starting the treatment, and the rollers were lubricated with ultrasound gel. Immediately after the first treatment session, the finger and the wrist flexor spasticity had reduced to 2/5 and 2/5 respectively on the modified Ashworth scale in both patients, and this effect lasted for about 15 minutes. Over 4 weeks of treatment 5 days/week, the distal tone reduction became persistent. Both patients had a modified Ashworth score of 2/5 for the fingers, tested while supine before the daily treatment session started, and the wrist scores were 2/5 (# 2) and 3/5 (#1). Passive hand care was easier, although active hand function did not change considerably; the FM scores were 9 and 13 respectively.

### Pilot study in sub-acute patients

The exploratory pilot study, approved by the local ethical committee, included eight hemiparetic patients who gave written informed consent. They suffered from a middle cerebral artery infarct 4–6 weeks earlier (additional file 1). The subjects were at least mobile in wheelchairs, and their Barthel Index (BI, 0–100) ranged from 55 to 70. Their upper limb was flaccid, and they could not volitionally extend the wrist or fingers: the FM score (0–66) ranged from 5 to 18. MRC grades of motor power are shown in additional file 3. The sensation was normal or only mildly affected, when tested for pain, touch, two-point discrimination, and position sense. All patients were already participating in a comprehensive in-patient rehabilitation programme including 45 min of physiotherapy and 30 min of occupational therapy every workday. The major treatment goals were restoration of stance and gait, and independence in ADL. About 15% of total therapy time was devoted to upper limb work, such as shoulder mobilisation, holding the paretic arm extended while lying, weight acceptance tasks over the fully extended arm and bilateral manoeuvres such as moving a duster on a table.

After consent, the patients were randomly allocated to two groups, A and B, by drawing a lot from an envelope. Both groups continued with conventional therapy. Group A had additional 20 minutes on the Finger Trainer each work day for 4 weeks, and group B had the same duration of daily group practice of bimanual upper limb exercises, in which the patient held a dusting cloth in their weak hand and pushed it over the surface of a table with their strong hand.

The assessments before and after the 4 week intervention included the FM (total upper limb 0–66 and total distal 0–30), the Box & Block test [19], a sum score of muscle power (0–30, wrist and finger flexion/extension and thumb abduction and adduction) based on the MRC (0–5), and a sum score of muscle tone (0–15, resistance to passive wrist and finger extension and thumb abduction, tested while supine) based on the modified Ashworth score (0–5). None of the highly paretic patients was able to transfer a block within the Box & Block test initially. The FM assessment (0–66) was videotaped with a mirror placed in an angle of 45° placed behind the patient, and an experienced therapist who was blinded with respect to the group assignment assessed & scored the videos of all patients.

## Results

In addition to their regular programme, the four A group patients practised with the Finger Trainer for 20 minutes every workday for four weeks. The cam shaft rotation ranged from 20 to 25 revolutions/minute, and the vibration frequency from 25 to 35 Hz. Therapy-related side effects did not occur. Results for the 8 sub-acute stroke patients are shown in additional file 2 and additional file 3. The mean distal Fugl-Meyer score increased in the control group from 1.25 > 2.75 (ns) and 0.75 > 6.75 in the treatment group (p < .05, paired t test vs baseline & t test vs control final scores). Median Modified Ashworth score increased in the control group, but not in the treatment group. The distal upper limb muscle strength improved to a similar degree (see additional file 3). Only one patient, in the treatment group, showed any improvement in active hand function, becoming able to transfer 16 blocks within one minute (additional file 2). He also used his paretic hand functionally in daily life, for instance when pulling off his pullover or holding objects, e.g. a toothpaste tube. He was not able to open the tube with his affected hand. The remaining seven subjects did not spontaneously use their affected hand. The Barthel Indices of all patients improved, and there was no apparent group difference (additional file 3). Subjectively, the four A group patients were positive about treatment with the Finger Trainer as they felt something was happening with their paretic hand and the asynchronous movement of the fingers in combination with the vibration felt comfortable.

## Discussion

The Finger Trainer is a newly developed device for the sensory-motor rehabilitation of the plegic fingers after stroke. This small study shows a clinically significant difference in spasticity in the treatment & control groups, which would require a larger series to test statistically. We were pleasantly surprised to find a statistically significant improvement in Fugl-Meyer score in such a small trial and this certainly justifies a larger study to give more conclusive results. Patients tolerate and even like using it. Side effects did not occur, although we avoided patients with pre-existing hand pain, arthritis or soft tissue problems, as we felt these people were most likely to develop problems. It remains to be seen if arthritis, which is very common among people in the age range who have strokes, is aggravated or helped by repetitive gentle movement.

The two chronic patients showed reduced resistance to passive finger movements. Prolonged immobilization of the joint could have resulted in changes in the soft tissue and joint compliance associated with developing contractures [16] and the repetitive passive movement of the fingers may have improved soft tissue compliance. The vibration could also have played a role: Ahlborg et al., for instance, reported a tone-diminishing effect on the knee extensors in adults with cerebral palsy following whole-body vibration [20].

None of the four sub-acute A group patients, but three B group patients, developed a clinically significant increase in the resistance to passive finger extension. This finding supports the recommendation of Pandayan et al. that passive movements around the joints in non-functional patients should begin very early during their rehabilitation programme to prevent contractures [16].

The fingers share one of the largest cortical representation areas in the primary motor area. Two studies using several complementary techniques have shown that passive limb movements, such as those made by the Finger Trainer, cause activation in the sensorimotor cortex in the same areas as active movements [21,22]. In healthy subjects, positron emission tomography has shown that active and passive elbow movements resulted in identical strong increases in regional blood flow in the sensorimotor cortex [21]. Similarly magnetencephalography has revealed dipolar sources within 1 cm of the central sulcus following passive finger movement [22].

While this evidence shows the benefit of passive movements, active movements do lead to greater cortical and muscle activation. Lotze et al measured changes in activation in the contralateral primary motor cortex (cM1) using fMRI and transcranial magnetic stimulation (TMS) following 30 min of either active or passive wrist extension in healthy subjects [23]. While passive movements caused some increase in activation, active training led to more prominent increases in fMRI activation, recruitment curves (TMS) and intracortical facilitation (TMS). The authors concluded that the results were consistent with the concept of a pivotal role for voluntary drive in motor learning. Accordingly, the finger trainer in its present form is rather limited as it only offers a passive movement, unlike the Rutgers Hand Master and the Howard Hand robot [10,12]. However, it is intended primarily for stroke patients with plegic fingers.

For this subgroup of severely affected patients, who are unable to actively move their fingers, sensory stimulation may be particularly important. Hummelsheim et al. reported that, compared to voluntary muscle activation, a similarly strong facilitation of movement was obtained with cutaneous and proprioceptive stimuli in severely affected patients [24]. In adult owl monkeys, Jenkins et al. reported that functional cortical remodelling of the S1 koniocortical field resulted from cutaneous stimulation of a limited sector of skin on the distal phalanges [25]. Byl et al. successfully used attended, graded, repetitive sensory and motor training activities, 1.5 hours per week for eight weeks, to improve the fine motor control and sensory discrimination tasks in chronic stroke patients [26]. Somatosensory stimulation, delivered via electrical stimulation, also positively influenced the sensorimotor recovery in chronic stroke patients [27,28].

To enhance the sensory stimulation provided by the Finger Trainer, strips with different surface texture were attached to the inner surface of the concave roll of the index finger, and the patients were instructed to discriminate the different textures. Secondly, vibration was applied to primarily activate the Paccini corpuscles of the finger tips. Since the vibration motor was under the cam shaft, the small amplitude vibration was not only felt in the distal phalanges but in the whole arm up to the shoulder. There is neurophysiological evidence in cats [29] and humans [30] that sensory stimulation induces long-term potentiation in the motor cortex, and increases corticospinal excitability. In clinical studies of stroke patients, Shirahashi et al. reported that vibratory stimulation on the hand facilitated voluntary movements of a hemiplegic upper limb [31]. Tihanyi et al. showed that one bout of whole body vibration transiently increased voluntary force and muscle activation of the quadriceps muscle affected by stroke [32].

In conclusion, the inexpensive Finger Trainer is a simple way of providing more intensive stimulation and passive stretching of plegic fingers after stroke. These preliminary results suggest further studies to examine its effect on muscle tone, ease of care, pain & active function are justified.

## Competing interests

The spouse of the first author owns the company, Reha-Stim, holding the national patent.

## Authors' contributions

SH conceived the device and study, and drafted the manuscript. HK manufactured the device. JW recruited, treated and assessed the patients. CT designed the device. SGBK helped with the design & analysis of the study and the preparation of the manuscript. All authors reviewed the final manuscript.

## Supplementary Material

Additional file 1 Table 1: Clinical data of both groups at study onset Click here for file

Additional file 3 Table 3: Individual and mean (SD) values of power & muscle tone of both groups at study onset and study end. Click here for file

Additional file 2 Table 2: Individual and mean (SD) values of movement & function of both groups at study onset and study end. Click here for file
