# Supplementary material for: A new electromechanical trainer for sensorimotor rehabilitation of paralysed fingers: A case series in chronic and acute stroke patients
Source: J Neuroeng Rehabil. 2008 Sep 4;5:21. doi: 10.1186/1743-0003-5-21 (PMC2542391; doi:10.1186/1743-0003-5-21)
Supplement: Additional file 1 — Table 1: Clinical data of both groups at study onset [file 1743-0003-5-21-S1.doc]

## Table 1: Clinical data of both groups at study onset

|  | **Experimental group** | | | | | **Control group** | | | | |
| --- | --- | --- | --- | --- | --- | --- | --- | --- | --- | --- |
| Clinical data | **Pat.1** | **Pat.2** | **Pat.3** | **Pat.4** | Mean (±SD) | **Pat.1** | **Pat.2** | **Pat.3** | **Pat.4** | Mean (±SD) |
| Diagnosis | ischemia | ischemia | ischemia | ischemia | - | ischemia | ischemia | ischemia | ischemia | - |
| Interval [weeks] | 5 | 6 | 4 | 4 | 4.8(±1.0) | 5 | 5 | 4 | 4 | 4.5(±0.6) |
| Hemiparesis | left | right | left | right | - | left | left | right | right | - |
| Sex | ♂ | ♂ | ♀ | ♂ | - | ♂ | ♀ | ♂ | ♀ | - |
| Age [years] | 56 | 53 | 72 | 62 | 60.8(±8.4) | 61 | 57 | 58 | 65 | 60.3(±3.6) |
| Barthel Index (0-100) | 55 | 70 | 60 | 65 | 62.5(±6.5) | 55 | 65 | 60 | 65 | 61.3(±4.8) |
| Aphasia | no | yes | no | yes | - | no | no | no | yes | - |
| Apraxia | no | no | no | yes | - | no | no | no | yes | - |
| Neglect | no | no | no | no | - | yes | no | no | no | - |
