# Supplementary material for: A new electromechanical trainer for sensorimotor rehabilitation of paralysed fingers: A case series in chronic and acute stroke patients
Source: J Neuroeng Rehabil. 2008 Sep 4;5:21. doi: 10.1186/1743-0003-5-21 (PMC2542391; doi:10.1186/1743-0003-5-21)
Supplement: Additional file 3 — Table 3: Individual and mean (SD) values of power & muscle tone of both groups at study onset and study end. [file 1743-0003-5-21-S3.doc]

**Table 3: Individual and mean (SD) values of power & muscle tone of both groups at study onset and study end.**

| **Dependent variables** | Experimental group | | | |  | **Control group** | | | |  |
| --- | --- | --- | --- | --- | --- | --- | --- | --- | --- | --- |
|  | **Pat.1** | **Pat.2** | **Pat.3** | **Pat.4** | **Mean (±SD)** | **Pat.1** | **Pat.2** | **Pat.3** | **Pat.4** | **Mean (±SD)** |
| **MRC distal Sum Score**  [0-30] **initial** | 1 | 9 | 4 | 5 | 4.8(±3.3) | 2 | 6 | 10 | 2 | 5.0(±3.8) |
| **MRC distal Sum Score**  [0-30] **final** | 9 | 20 | 14 | 12 | 13.8(±4.6) | 2 | 11 | 12 | 4 | 7.3(±5.0) |
| **MRC wrist**  [0-5] flexion/extension **initial** | 1/0 | 1 | 2 | 2 | 1.75(±0.5)/ 1.0(±0.82) | 2/0 | 1 | 1 1/2 | 0/0 | 1.75(±1.26)/ 1.0(±1.15) |
| **MRC wrist** [0-5] flexion/extension **final** | 1 | 1 | 1 1/3 | 1 1/2 | 3.25(±0.96)/ 2.75(±0.96) | 2/0 | 1 | 1 | 2/0 | 2.5(±0.58)/ 1.5(±1.73) |
| **MRC finger** [0-5] flexion/extension **initial** | 0/0 | 1 | 1/0 | 2/0 | 01.25(±0.96)/ 0.5(±1.0) | 0/0 | 2/0 | 1 1/2 | 2/0 | 1.75(±1.23)/ 0.5(±1.0) |
| **MRC finger** [0-5] flexion/extension **final l** | 1 | 1 | 1 1/2 | 1 1/2 | 2.75(±0.5)/ 2.25(±0.5) | 0/0 | 2 | 1 1/2 | 2/0 | 1.75(±1.23)/ 0.75(±0.96) |
| **MRC thumb** [0-5] adduction/abduction **initial** | 0/0 | 1/0 | 0/0 | 0/0 | 0.25(±0.5)/ 0.0(±0.0) | 0/0 | 0/0 | 0/0 | 0/0 | 0.0(±0.0)/ 0.0(±0.0) |
| **MRC thumb** [0-5] adduction/abduction **final** | 1/0 | 1 | 2/0 | 2/0 | 2.0(±0.82)/ 0.75(±1.5) | 0/0 | 1 | 1/0 | 0/0 | 0.5(±0.58)/ 0.25(±0.5) |
| **Modified Ashworth distal Sum** **Score** **initial** [0-15] | 0 | 1 | 0 | 0 | 0.25(±0.5) | 5 | 2 | 0 | 0 | 1.75(±2.36) |
| **Modified Ashworth distal Sum Score final** [0-15] | 0 | 0 | 0 | 0 | 0.0(±0.0) | 10 | 5 | 0 | 5 | 5.0(±4.08) |
| **Modified Ashworth** **Score**  [0-5] **initial**  *wrist-finger-thumb* | 0-0-0 | 1-0-0 | 0-0-0 | 0-0-0 | 0.25(±0.5) 0.0(±0.0) 0.0(±0.0) | 2-2-1 | 1-1-0 | 0-0-0 | 0-0-0 | 0.75(±0.96) 0.75(±0.96) 0.25(±0.5) |
| **Modified Ashworth** **Score** [each 0-5] **final**  *wrist-finger-thumb* | 0-0-0 | 0-0-0 | 0-0-0 | 0-0-0 | 0.0(±0.0) 0.0(±0.0) 0.0(±0.0) | 3-4-3 | 2-2-1 | 0-0-0 | 2-2-1 | 1.75(±1.23) 2.0(±1.63) 1.25(±1.23) |
