# Supplementary material for: A new electromechanical trainer for sensorimotor rehabilitation of paralysed fingers: A case series in chronic and acute stroke patients
Source: J Neuroeng Rehabil. 2008 Sep 4;5:21. doi: 10.1186/1743-0003-5-21 (PMC2542391; doi:10.1186/1743-0003-5-21)
Supplement: Additional file 2 — Table 2: Individual and mean (SD) values of movement & function of both groups at study onset and study end. [file 1743-0003-5-21-S2.doc]

**Table 2: Individual and mean (SD) values movement & function of both groups at study onset and study end.**

| **Dependent variables** | Experimental group | | | |  | **Control group** | | | |  |
| --- | --- | --- | --- | --- | --- | --- | --- | --- | --- | --- |
|  | **Pat.1** | **Pat.2** | **Pat.3** | **Pat.4** | **Mean (±SD)** | **Pat.1** | **Pat.2** | **Pat.3** | **Pat.4** | **Mean (±SD)** |
| **Fugl-Meyer Score** (FM; 0-66) **initial** | 5 | 18 | 10 | 12 | 11.3(±5.4) | 5 | 17 | 12 | 8 | 10.5(±5.2) |
| **Fugl-Meyer Score** (FM; 0-66) **final** | 19 | 51 | 19 | 27 | 26.5(±10.4) | 10 | 30 | 20 | 14 | 18.5(±8.7) |
| **FM** – *proxima*l (0-36) **initial** | 5 | 6 | 10 | 11 | 10.5(±4.5) | 5 | 14 | 10 | 8 | 9.3(±3.8) |
| **FM** – *proximal* (0-36) **final** | 12 | 31 | 15 | 21 | 19.8(±8.4) | 10 | 24 | 16 | 13 | 15.8(±6.0) |
| **FM** – *distal* (0-30) **initial** | 0 | 2 | 0 | 1 | 0.8(±1.0) | 0 | 3 | 2 | 0 | 1.3(±1.5) |
| **FM** – *distal* (0-30) **final** | 7 | 10 | 4 | 6 | 6.8(±2.5) | 0 | 6 | 4 | 1 | 2.8(±2.8) |
| **Box&Block** [n] **initial** | 0 | 0 | 0 | 0 | 0.0(±0.0) | 0 | 0 | 0 | 0 | 0.0(±0.0) |
| **Box&Block** [n] **final** | 0 | 16 | 0 | 0 | 4.0(±8.0) | 0 | 0 | 0 | 0 | 0.0(±0.0) |
| **Barthel Index** (0-100) **initial** | 55 | 70 | 60 | 65 | 62.5(±6.5) | 55 | 65 | 60 | 65 | 61.3(±4.8) |
| **Barthel Index** (0-100) **final** | 70 | 90 | 80 | 80 | 80.0(±8.2) | 65 | 90 | 75 | 75 | 76.3(±10.3) |
